# Peptidome analysis of human intrauterine adhesion tissues and the identification of antifibrotic peptide

**DOI:** 10.7555/JBR.36.20220059

**Published:** 2022-07-28

**Authors:** Xiangdong Hua, Yan Zhang, Juan Xu, Lu Xu, Yaqian Shi, Dazhen Yang, Xiaoyan Gu, Sumin Wang, Xuemei Jia, Feng Xu, Jie Chen, Xiaoyan Ying

**Affiliations:** 1 Department of Gynecology, Women's Hospital of Nanjing Medical University, Nanjing Maternity and Child Health Care Hospital, Nanjing, Jiangsu 210004, China; 2 Department of Obstetrics and Gynecology, the Second Affiliated Hospital of Nanjing Medical University, Nanjing, Jiangsu 210011, China

**Keywords:** Asherman's syndrome, fibrosis, peptide, stromal cells

## Abstract

Intrauterine adhesion (IUA) is a common clinical endometrial disease, which can severely damage the fertility and quality of life in women. This study aims to find the differentially expressed endogenous peptides and their possible roles in IUA. Liquid chromatography-mass spectrometry was used to identify the peptidomic profiling of IUA tissues, and the differentially expressed peptides were screened out. Using real-time quantitative PCR, Western blotting, and immunocytochemistry staining, the function of six endogenous peptides was verified *in vitro*. It was found that peptide 6 (T6) (peptide sequence: TFGGAPGFPLGSPLSSVFPR) could inhibit the expression of TGF-β1-induced cell fibrosis in human endometrial stromal cell line and primary human endometrial stromal cell at a concentration of 50 μmol/L. This study provides new targets for further clarifying the formation and prevention of IUA.

## Introduction

Intrauterine adhesion (IUA), known as Asherman's syndrome, may cause hypomenorrhea or amenorrhea, periodic hypogastralgia, infertility, early embryonic death, and even habitual abortion^[1]^, which can severely damage the fertility and quality of life in women. Therefore, it is of great importance to reduce the occurrence of IUA and improve the physiology and reproductive ability of patients in clinical practice.

The clinical pathological presentation of IUA is mainly marked by fibrosis of the endometrium, while the treatment of IUA focuses on the removal of adhesive tissues, prevention of re-adhesion, and promotion of endometrial regeneration^[2]^. For patients suffering from severe adhesions, the current treatment methods are unsatisfactory. Lack of normal endometrium, poor intrauterine environment, and easy relapse of adhesions mean that successful natural conception or embryonic transplantation is still challenging for patients with IUA^[3]^. Many efforts have been made to restore and regenerate the endometrium through marrow mesenchymal and endometrial or embryonic stem cell transplantation. Several cases of successful conception have been reported after stem cell transplantation^[4–6]^, but the low survival rate, difficulty in induced directional differentiation, and tumorigenicity of stem cells remain to be resolved before clinical application.

No effective method is available for the clinical prevention and treatment of IUA, especially in severe cases. Prevention of endometrial fibrosis is the precondition for promoting endometrial regeneration^[7]^, so it is important to prevent, inhibit, and even reverse endometrial fibrosis. The main pathogenic mechanism related to IUA is the active proliferation of fibrocytes, *i.e.*, endometrial basal layer damage due to any condition might lead to the generation of various fibrosis factors, stimulating the proliferation of fibroblasts and the excessive deposition of extracellular matrix (ECM), ultimately leading to the proliferation of fibrous connective tissues and the formation of scars^[8]^. Studies on endometrial fibrosis have covered multiple areas such as cytokines (*e.g.*, transforming growth factor beta-1 (TGF-β1)^[9–10]^, connective tissue growth factor (CTGF)^[11]^ and nuclear factor kappa-B (NF-κB)^[12]^), ECM-related factors (*e.g.*, matrix metallopeptidase 9 (MMP-9)^[13]^ and tissue inhibitor of metalloproteinase 1 (TIMP-1)^[14]^), and noncoding RNA (*e.g.*, miR-29b^[9]^ and miR-543^[15]^). Despite significant progress in those areas, no effective clinical application is known to date. How to decrease or reverse endometrial fibrosis and find a safe and effective prevention method in the clinic remains a challenge in gynecology.

Many bioactive peptides with important functions have been discovered, some of which are often used for medical purposes. At present, peptides have been widely used in the treatment of cancer, metabolic disorders, cardiovascular diseases, diabetes, and other diseases^[16]^. Because of their low toxicity and highly specific targeting, chemical modification can control the peptides' half-lives, giving them unique advantages like no other medicines^[17]^. Therefore, it is important to study the etiology of IUA from the perspective of peptides involvement and explore biologically active peptides that may be used in treatments. In addition, some endogenous peptides have been considered important regulators of fibrosis. For example, decorin, one of the ECM matrix components, can form different peptides under different pathogenic conditions, among which the peptides at the C-terminal 335–359 area can bind with CTGF and hinder the formation of fibrosis, while those at the N-terminal 42–71 area can restrain the activity of Smad2/3 by inhibiting the myostatin in a dose-dependent manner^[18]^. The antifibrotic polypeptide AcSDKP that naturally exists in plasma can inhibit the Smad signaling pathway in mesangial cells^[19]^. Zhang *et al* verified that a peptide (SPFYLRPPSF) from Cryab can ameliorate myocardial fibrosis *in vitro* and *in vivo*^[20]^. Moreover, N-acetyl-seryl-aspartyl-lysyl-proline, an endogenous peptide has been recognized as a valuable antifibrotic peptide in recent two decades^[21]^. These results showed that functional peptide molecules participated in regulating the formation of fibrosis.

Therefore, this study focused on profiling endogenous peptides from the IUA tissues and normal endometrial tissues in the proliferative stage using mass spectrometry, in the hope of providing new insights into the diagnosis, treatment, and prevention of IUA.

## Materials and methods

### Sample collection

The samples were collected from patients at the Women's Hospital of Nanjing Medical University, including IUA tissues from patients without fertility requirements (referring to the classification criterion of the American Fertility Society, 1988^[22]^) and the normal endometrial tissues in the proliferative stage from patients with panhysterectomy after being diagnosed with cervical lesions. There were three cases in each group and samples were collected using scissors. No complication was observed in all cases. Details of the patients are shown in ***Table 1***. The study obtained the approval of the Ethics Committee of the Women's Hospital of Nanjing Medical University (Approval No. 2017KY-022). All the patients were informed about the research, and written informed consent was obtained. The tissues were around 5 mm × 5 mm × 5 mm and were collected immediately after surgery, quickly frozen in liquid nitrogen, and stored at −80 °C until use.

**Table 1:** Detailed clinical information of patient samples

Patient	Age (years)	Disease	Stage	Sample tissues
1	38	Intrauterine adhesion	Severe	Intrauterine adhesions
2	40	Intrauterine adhesion	Severe	Intrauterine adhesions
3	45	Intrauterine adhesion	Severe	Intrauterine adhesions
4	42	Cervical intraepithelial neoplasia	Ⅲ	Endometrial tissues
5	44	Cervical intraepithelial neoplasia	Ⅲ	Endometrial tissues
6	45	Cervical intraepithelial neoplasia	Ⅲ	Endometrial tissues

### Peptide extraction

The samples were placed in a mortar and ground into a powder in liquid nitrogen. Then, the powder was placed in a tube, followed by lysing with 50% protein lysis buffer (lysate buffer: 8 mol/L urea, 100 mmol/L TEAB, 1 mmol/L PMSF, 2 mmol/L EDTA, pH 8.0) and incubating on ice for 5 minutes. Then, dithiothreitol (DTT) with a final concentration of 10 mmol/L was added to the lysate, followed by ice-bath ultrasonic treatment for 15 minutes. The lysate was centrifuged at 13 000 *g*, 4 °C for 30 minutes, and the supernatant was collected in a new centrifuge tube. Next, DTT was added to the centrifuge tube to reach a final concentration of 10 mmol/L, and then the sample was incubated in a 56 °C water bath for 30 minutes for a reduction reaction. Later, iodoacetamide was added to reach a final concentration of 55 mmol/L, and the mixture was placed in the dark at room temperature for 30 minutes for the alkylation reaction. Then, the sample was transferred to a 10 kDa ultrafiltration tube and centrifuged at 10 000 *g*, 4 °C for 30 minutes, and the flow-through (peptide samples) was collected.

### Liquid chromatography/mass spectrometry

Peptide identification was performed using an Eksigent nanoLC system (SCIEX, USA) coupled with a TripleTOF 5600 system. The peptide samples were dissolved in a mixture of 2% acetonitrile and 0.1% formic acid and then analyzed using an Eksigent nanoLC system coupled with a TripleTOF 5600 system. The peptide samples were added onto a C18 trap column (5 μm, 100 μm × 20 mm), and gradient elution was performed using a C18 separation column (3 μm, 75 μm × 150 mm) in a 90-minute time gradient at 300 nL/min flow speed. The two mobile phases were buffer solution A (composed of 2% acetonitrile, 0.1% formic acid) and buffer solution B (composed of 98% acetonitrile, 0.1% formic acid). For information-dependent acquisition, first-order mass spectrogram scanning was conducted with 250 milliseconds ion accumulation time, and the second-order mass spectrogram of 30 precursor ions was collected with 50 milliseconds ion accumulation time. MS1 and MS2 spectra were collected within the range of 350–1500 m/z and 100–1500 m/z, respectively. The dynamic exclusion time for precursor ions was set as 15 seconds.

### Liquid chromatography/mass spectrometry data analysis

*Homo sapiens* protein sequences in the UniProt database containing 20 240 protein sequences were included in this study (UniProt reference was downloaded until August 18, 2018). The mass spectrometry data were searched in the database by using ProteinPilot software, and nonlabeled peptides/proteins were quantified using Skyline v3.7 software (MacCoss Lab, USA).

Filtration standard: for protein and peptide identification, the unused protein score is ≥1.3; the peptide fragment reliability is above 95% (false discovery rate <1%); and the identified protein contained at least one unique peptide fragment.

The identified peptides were quantitatively analyzed using Skyline v3.7 software. The peptide fragments were mapped onto precursor proteins for quantification, and the proteins with significant differences were identified.

Peptide function annotation: Gene ontology (GO) with three ontologies (molecular function, cellular component, and biological process), cluster of orthologous groups of proteins (COG), and Kyoto encyclopedia of genes and genomes (KEGG) databases were used for the functional identification of differentially expressed peptides. Enrichment analysis of the GO function can provide information about GO function entries with significant enrichment by comparing them with the identified protein background. Then, the differential peptide precursors that are significantly correlated with specific biological functions can be determined. When the abundance ratio of a peptide with a folding change is ≥1.5, that peptide can be deemed as a differential peptide among different samples. In the analysis, all differential peptides were mapped onto the terms in the GO database ( http://www.geneontology.org/). Then, hypergeometric verification was used to calculate the *P*-value, and *P*-value <0.05 was taken as the threshold of significance. GO terms that satisfy this condition would be defined as GO terms with significant enrichment among differential peptide precursors. Through GO significance analysis, the major biological functions of differential peptides can be determined.

The identified peptide precursors can be compared with the COG database to predict the possible functions of the peptides and to conduct functional classification statistics. KEGG pathway analysis was used for the pathway enrichment of differentially expressed peptides.

By screening differentially expressed peptides located in the functional domain, the sequence of precursor proteins of differentially expressed peptides was imported into the SMART database ( http://smart.embl-heidelberg.de/). The functional domains of precursor proteins were identified, and the differentially expressed peptide sequences were compared with the functional domain sequences to evaluate whether the differentially expressed peptide sequences were located on the functional domain of precursor proteins.

### Peptide synthesis and dissolution

The screened peptides used in the experiment were synthesized by GenScript (China), with a purity of ≥95%, and all of them were standard trans acetate salt and unmodified. The peptides were dissolved in sterilized water at 20 mmol/L and diluted to 10 μmol/L, 20 μmol/L, 50 μmol/L, and 100 μmol/L according to the experimental requirements.

### Cell culture

Human endometrial stromal cells (hESCs) were purchased from Shanghai Xin Yu Biotech Co., Ltd. (China) and cultured in Dulbecco's Modified Eagle's Medium/Ham's F12 mixture (DMEM/F12) (Gibco, USA) plus 10% fetal bovine serum (FBS, Gibco) and penicillin-streptomycin solution (Sigma, USA; 1:100), at 37 °C, 5% CO_2_, and saturated humidity. When the cell density reached 80%–90%, the cells were sub-cultured according to a ratio of 1:3–1:4, and the cells in the logarithmic growth phase were taken for subsequent experiments.

Isolation and culture of primary hESCs (phESCs): Full-thickness human endometrial tissue samples were collected and kept in phosphate-buffered saline (PBS, Gibco) plus penicillin-streptomycin solution (1:100) at 4 °C, and subjected to cell isolation within 30 minutes. The tissue samples were minced into 0.5–1 mm^3^ pieces, and were incubated in DMEM/F12 supplemented with 0.2% collagenase Ⅰ (Sigma) for 60 minutes at 37 °C. The dispersed endometrial tissue fragments/cells were filtered sequentially through sterile 40–100 μm nylon strainers (BD Biosciences, USA) to remove undigested tissue clumps and epithelial cells. The suspension was centrifuged at 1000 *g* for 5 minutes and the supernatant was discarded. Then the phESCs were resuspended in DMEM/F12 supplemented with 10% FBS and penicillin-streptomycin solution (1:100) and cultured at 37 °C, 5% CO_2_ and saturated humidity. Cells were supplemented with fresh culture medium every 3 days until they were confluent. Primary cells were identified by immunofluorescence of vimentin (***Supplementary Fig. 1***, available online). The 3^rd^ to 6^th^ passages were used for subsequent experiments. The TGF-β1 (5 ng/mL; R&D Systems, USA) treated hESCs or phESCs were cocultured with the peptides. After incubation for 48 hours, the total RNA of each group was extracted for real-time quantitative PCR (RT-qPCR) analysis. After incubation for 72 hours, the total protein of each group was extracted for Western blotting, the cells were selected for immunocytological experiment.

### Immunocytochemistry

The hESCs were seeded in 24-well plates and fixed with 4% polyformaldehyde for 20 minutes, permeabilized with 0.5% Triton X-100 for 20 minutes. Then we used 0.3% hydrogen peroxide for 10 minutes to inactivate endogenous peroxidase. Goat serum was added at room temperature for 30 minutes. The primary antibody anti-COL1A1 (type Ⅰ collagen, Cell Signaling Technology, USA; 1:200 dilution) was incubated for 2 to 3 hours. The second antibody dilution was added and incubated at room temperature for 1 hour. Finally, the cells were stained with 3,3-diaminobenzidine and counterstained with hematoxylin for 5 minutes.

### Immunofluorescence assay

The hESCs or phESCs were seeded in 24-well plates and fixed with 4% polyformaldehyde for 20 minutes, permeabilized with 0.5% Triton X-100 for 20 minutes, and blocked with goat serum for 30 minutes. The cells were then incubated overnight with primary antibodies such as anti-α-SMA (alpha-smooth muscle actin; Proteintech, China; 1:200 dilution) or anti-COL1A1 (1:200 dilution) at 4 °C. The next day, secondary antibody Alexa Fluor 555-conjugated goat anti-rabbit IgG (Cell Signaling Technology; 1:1000 dilution) or Alexa Fluor 488-conjugated goat anti-rabbit IgG (Servicebio, China; 1:200 dilution) was incubated at room temperature for 1 hour in the dark, and the nuclei were stained with DAPI (Servicebio; 2 μg/mL). The image was observed under a fluorescence microscope (Zeiss, Germany).

### Real-time quantitative PCR

The cells were collected and total RNA was extracted according to the manufacturer's protocol of the RNA Extraction and Purification Kit (Thermo, USA). 1 μg total RNA was reverse-transcribed into cDNA (Vazyme, China). RT-qPCR was performed using ViiA 7 RT-qPCR system (Life Technologies, USA). *ACTB* was used as the internal reference and each experiment was repeated 3 times. The primers were synthesized by Sangon Biotech (Shanghai, China) and the sequences are shown in ***Table 2***.

**Table 2:** Specific information of primers for real-time quantitative PCR

Gene names	Forward primer sequence (5′→3′)	Reverse primer sequence (5′→3′)
*ACTA2*	AAAGACAGCTACGTGGGTGACGAA	TTCCATGTCGTCCCAGTTGGTGAT
*COL1A1*	AGCCAGCAGATCGAGAACAT	TCTTGTCCTTGGGGTTCTTG
*CTGF*	ACCGACTGGAAGACACGTTTG	CCAGGTCAGCTTCGCAAGG
*FN1*	ACAACCCCTACAAACGGCCA	TAGTCAATGCCCGGCTCCAG
*VIM*	TGCCGTTGAAGCTGCTAACTA	CCAGAGGGAGTGAATCCAGATTA
*CDH2*	TTTGATGGAGGTCTCCTAACACC	ACGTTTAACACGTTGGAAATGTG
*ACTB*	TCCCTGGAGAAGAGCTACGA	AGCACTGTGTTGGCGTACAG

### Western blotting

After extracting the total protein of cells, the protein concentration was measured by BCA assay. The protein samples and markers were added to the SDS-PAGE gel in sequence. After electrophoresis, the methanol soaked PVDF membrane (Bio-Rad, USA) was covered on the gel surface, and the membrane was continuously transferred at 400 mA for 90 minutes, then blocked with 5% skim milk at room temperature for 2 hours and incubated overnight with primary antibodies such as anti-α-SMA (1:1000 dilution), anti-COL1A1 (1:1000 dilution) and GAPDH (Proteintech; 1:20 000 dilution) at 4 °C. Secondary antibodies were incubated at room temperature for 1 hour the next day. Imaging was performed by chemiluminescence imaging system (Tanon, China). Finally, Image J was used to quantify the strip density. We used GAPDH as the internal reference.

### Statistical analysis

All cell experiments were repeated three times. Statistical analyses were conducted using GraphPad Prism 7.0 (GraphPad Software, USA). The data in accordance with normal distribution are presented as mean ± standard deviation. Statistical analyses were performed using Student's *t*-test. *P*-value <0.05 and fold change ≥1.5 were considered statistically significant.

## Results

### Identification of proteins and peptides

**Figure 1:**
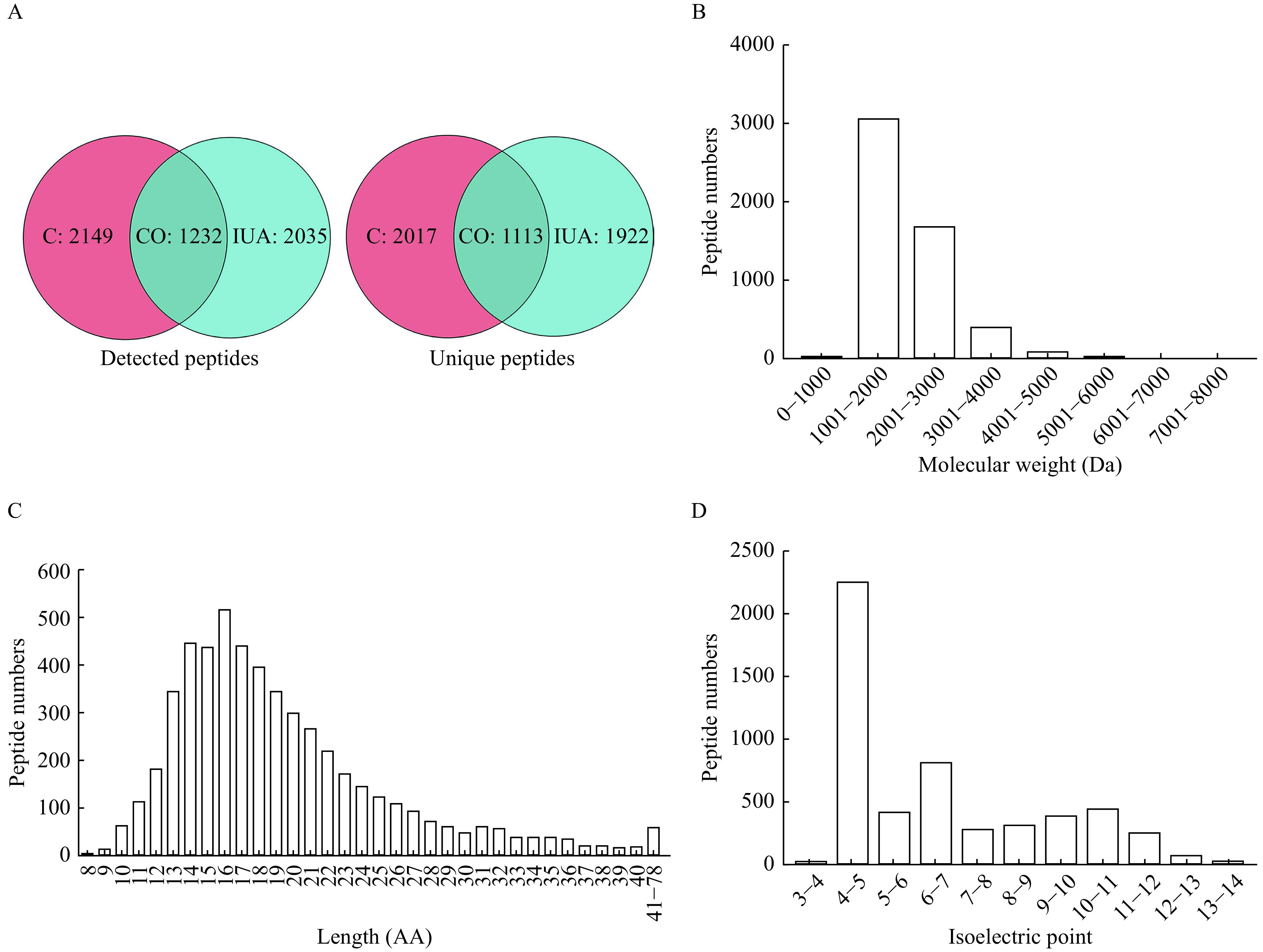
Characteristics of the peptidomic profiling of the intrauterine adhesion and normal endometrial tissues.

A total of 5191 peptides from 784 precursor proteins were identified by liquid chromatography/mass spectrometry, and the peptide numbers in IUA tissues and endometrial tissues in the normal proliferative stage were 1411±370 and 1866±57, respectively. The numbers of detected peptides and unique peptides in both IUA tissues and endometrial tissues at the normal proliferative stage were 1232 and 1113, respectively (***Fig. 1A***). The molecular weight of the peptides mainly ranged between 1000 Da and 4000 Da (***Fig. 1B***). Most of the identified peptides were 10 to 30 amino acids in length, while the average length was 19.25 amino acids (***Fig. 1C***). The isoelectric points of peptides ranged between 3 and 14, and more than 50% of isoelectric points of peptides ranged between 4 and 5 (***Fig. 1D***).

### Quantitative analysis of differentially expressed peptides

**Figure 2:**
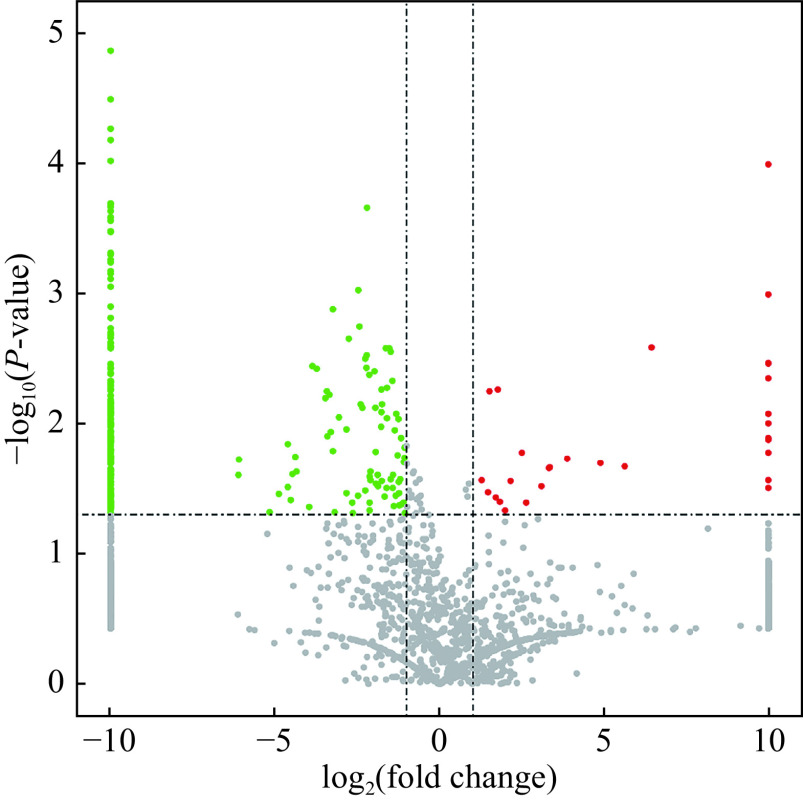
Volcanic map of differentially expressed peptides in intrauterine adhesion and normal endometrial tissues.

A total of 321 differentially expressed peptides from 155 precursor proteins were identified (***Fig. 2*** and ***Table 3***). Compared with the endometrial tissues in the normal proliferative stage, a total of 70 peptides from 56 precursor proteins were significantly upregulated, and 251 peptides from 120 precursor proteins were significantly downregulated in the IUA tissues (*P* <0.05, fold change ≥1.5).

**Table 3:** Differentially expressed peptides in IUA and endometrial tissues

Peptide sequences	Precursor proteins^a^	C_ Abund^b^	IUA_ Abund^c^	IUA/C	IUA/C *P*−value	Diff^d^
Top 10 down-regulated peptides						
HLPAEFTPAVHASLDK	HBA	1 078 293	3790	0.015	0.025	down
AAHLPAEFTPAVHASLDK	HBA	240 661	10 947	0.034	0.004	down
IEVAQANDIISSTEISSAEKVA	MAP4	57 435	1897	0.041	0.009	down
WGKVNVDEVGGEAL	HBB	4 933 448	583 759	0.044	0.039	down
WGKVNVDEVGGEALG	HBB	34 909 722	2 217 317	0.049	0.016	down
VDPENFRLLGNVL	HBD	1 077 313	104 362	0.065	0.016	down
HLPAEFTPAVHASLDKFLASVST	HBA	2 389 040	184 961	0.100	0.003	down
HVDPENFRLLGNVL	HBD	939 995	105 034	0.121	0.041	down
ELRPTLNELGISTPEELGLDK	COX5A	62 188	9155	0.211	0.037	down
LVTLAAHLPAEFTPAVHASLDKF	HBA	630 064	152 490	0.219	0.012	down
Top 10 up-regulated peptides						
TFGGAPGFPLGSPLSSPVFPR	DESM	14 955	1 283 394	85.818	0.003	up
HLPAEFTPAVHASLDKFLASVSTVLTSKYR	HBA	446	21 679	48.608	0.021	up
LTPEEKSAVTALWGKVNVDEVGGEALGRL	HBB	22 575	329 059	14.576	0.019	up
PEEKSAVTALWGKVNVDEVGGEALGRL	HBB	14 648	148 200	10.117	0.022	up
GKVGAHAGEYGAEALERM	HBA	52	521	9.933	0.022	up
VHLTPEEKSAVTAL	HBB	14 345	121 357	8.460	0.030	up
PGHLQEGFGCVVTNRFDQL	PAIRB	35 530	156 900	4.416	0.028	up
ITVLSAMTEEAAVAIKAMAK	IF5A1	56	219	3.939	0.047	up
VDEVGGEALGRL	HBB	32	118	3.652	0.013	up
VDETNMYEGVGRMF	PFD1	6893	24 362	3.534	0.040	up
Top 10 abundant unique peptides^e^						
VTIAQGGVLPNIQAVLLPK	H2A2B	0	9 682 910	999.000	–	up
GLPRQVYDPKYCLTPEYPELGEPAHNH HAHN	CNN1	0	358 204	999.000	–	up
STGGISVPGPMGPSGPR	CO1A1	0	205 592	999.000	–	up
GYPGNIGPVGAAGAPGPHGPVGPAGK	CO1A2	0	153 524	999.000	–	up
AAPRPSPAISVSVSAPAFYAPQK	ZYX	0	222 728	999.000	–	up
GVAGALRPLVQATVPATPEQPVLDLKRPF	UCRIL	0	457 181	999.000	–	up
SVELEEALPVTTAEGMAKKVTK	H1X	0	165 630	999.000	–	up
REPGYTPPGAGNQNPPGMYPVTGPK	LPP	0	94 975	999.000	–	up
FELFPSLSHNLLVD	DNPEP	0	67018	999.000	–	up
QFIAAQNLGPASGHGTPASSPSSSSLPSP MSPTPR	PALLD	0	165 446	999.000	–	up
Top 10 abundant unique peptides^f^						
HLPAEFTPAVHASLDKFLAS	HBA	7 119 940	0	−999.000	–	down
EAIPMSIPPEVKFNKPF	A1AT	579 548	0	−999.000	–	down
HLPAEFTPAVHASL	HBA	633 069	0	−999.000	–	down
ILDLGNNIHQWC	GELS	400 510	0	−999.000	–	down
FSEGCAPGSKKDSSLCKLCMGSGLNLCE PNNKEGYYGYTGAFR	TRFE	387 758	0	−999.000	–	down
LWGKVNVDEVGGEALG	HBB	251953	0	−999.000	–	down
SYDRAITVFSPDGHLF	PSA7	217 133	0	−999.000	–	down
GSGLNLCEPNNKEGYYGYTGAFR	TRFE	210 258	0	−999.000	–	down
AHLPAEFTPAVHASLDKFLAS	HBA	217 358	0	−999.000	–	down
SKPHSEAGTAFIQTQQLHAAM	KPYM	143 529	0	−999.000	–	down
^a^Precursor proteins showed with UniProt entry names; ^b^quantitative value of endometrial tissue, *n*=3; ^c^quantitative value of intrauterine adhesion tissue, *n*=3; ^d^IUA/C difference; ^e^the top 10 abundant peptides that specifically expressed in the intrauterine adhesion tissues; ^f^the top 10 abundant peptides that specifically expressed in the normal endometrial tissues. IUA: intrauterine adhesion.						

### Cleavage analysis of differentially expressed peptides

**Figure 3:**
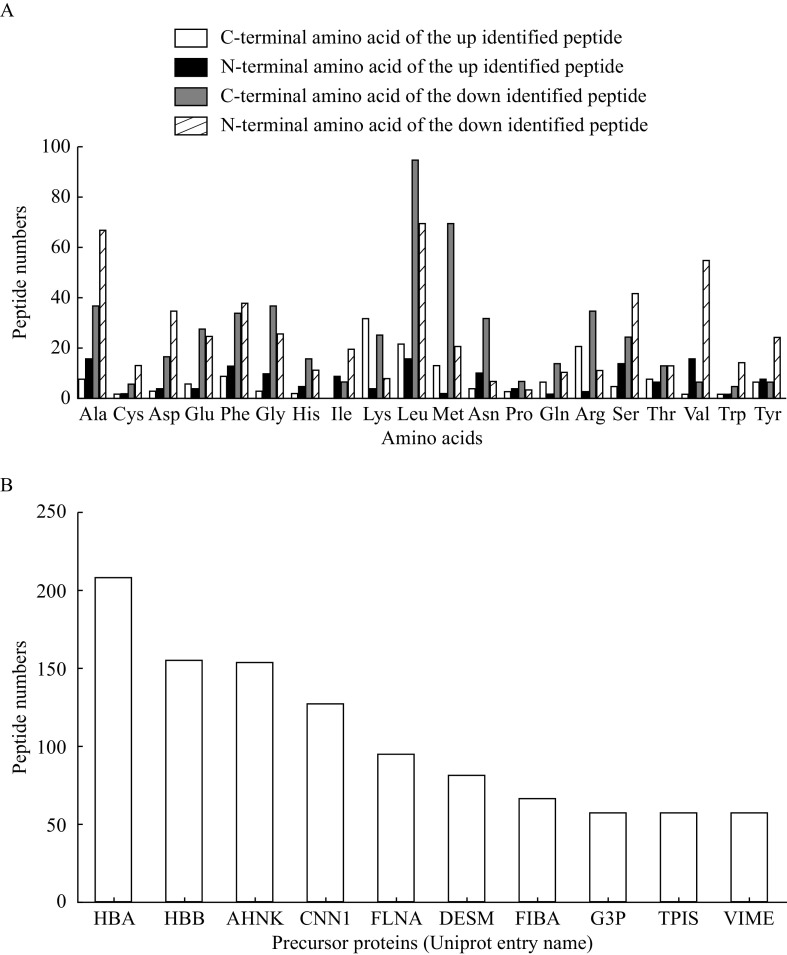
Cleavage analysis of differentially expressed peptides.

Endogenous peptides are often the degradation products of endogenous precursor proteins. Therefore, the degradation activity in the IUA tissues and endometrial tissues at the normal proliferative stage was further analyzed. Among the 321 differentially expressed peptides obtained from 155 precursor proteins, leucine (Leu) accounted for the largest proportion at the C-terminal of peptides, while among the differential peptides, alanine (Ala) accounted for the most proportion at the N-terminal of peptides. Among the up and down regulated peptides, the most significant difference was Leu and methionine (Met) at the C-terminal of peptides while Leu and Ala at the N-terminal of peptides (***Fig. 3A***). The top ten identified proteins containing the largest number of unique peptides are shown in ***Fig. 3B***.

### GO and pathway analysis of precursor proteins of differentially expressed peptides

**Figure 4:**
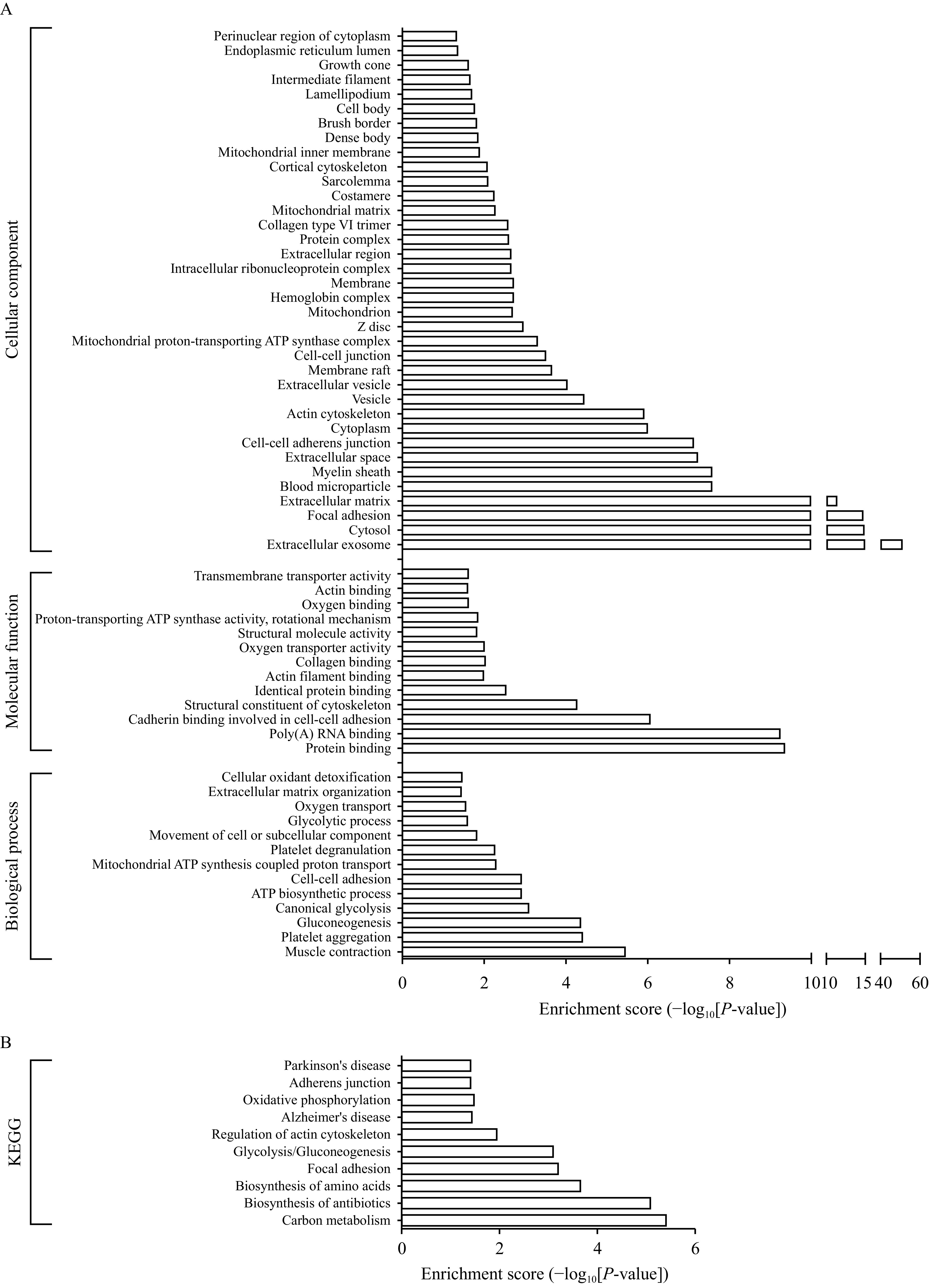
Gene Ontology and pathway analysis of precursor proteins of differentially expressed peptides.

GO and pathway annotations were conducted for the precursor proteins of differentially expressed peptides. GO annotation indicated that the top three annotations of molecular function are protein binding, poly (A) RNA binding, and cadherin binding involved in cell-cell adhesion; the top three annotations of cellular component are extracellular exosome, cytosol, and focal adhesion; the top three annotations of biological process are muscle contraction, platelet aggregation, and gluconeogenesis (***Fig. 4A***). KEGG pathway annotation indicates that the precursor proteins of differentially expressed peptides mainly participate in the metabolic pathways, focal adhesion, and the regulation of actin cytoskeleton (***Fig. 4B***).

### Screening of differentially expressed peptides located in functional domains

Functional domains are closely related to the physiological function of proteins, and several peptides are located in the functional domain of their precursor proteins and function as a part of their precursor proteins or as an antagonist of their precursor proteins^[23–24]^. In this study, by using the UniProt database, 101 differentially expressed peptide sequences from 48 precursor proteins were found to be located on their functional domains (***Supplementary Table 1***, available online). Bioinformatics analysis indicated that 23 of the 48 precursor proteins (UniProt entry names: PGK1, A1A, APOA1, ALBU, GELS, PTMA, ENOA, ALDOC, MIF, MIME, FLNA, COF, ETF, MDHC, LUM, PRELP, ITA1, TPIS, TAGL, DCXR, DESM, SYUA, and PPIA) were related to the fibrosis of various tissues, while 220 differentially expressed peptide sequences obtained from 121 precursor proteins were not located on their functional domains.

### Further screening of differentially expressed peptides

Since no suitable method could be used to predict the function of peptide, we first assumed that its function was related to its precursor protein. Through bioinformatics analysis, the differentially expressed peptides were further screened. Six endogenous peptides were selected because their precursor proteins were reported to be most closely related to fibrosis. Among them, peptide 1 (T1), peptide 2 (T2), peptide 3 (T3), and peptide 4 (T4) were downregulated in IUA tissues (*P*<0.05). Peptide 5 (T5) and peptide 6 (T6) were upregulated in IUA tissues (*P*<0.05). The detailed information about the peptides is shown in***Table 4***.

**Table 4:** Peptides selected by mass spectrometry and bioinformatics analysis

Peptide name	Peptide sequence	Length (AA)	Precursor proteins^a^
T1	YLDHNALESVPLN	13	sp|P20774|MIME_HUMAN
T2	YLDNNKISNIPDE	13	sp|P51884|LUM_HUMAN
T3	HVVPDQLMAFGGSSEP	16	sp|P14174|MIF_HUMAN
T4	WGKVNVDEVGGEAL	14	sp|P68871|HBB_HUMAN
T5	VDETNMYEGVGRMF	14	sp|O60925|PFD1_HUMAN
T6	TFGGAPGFPLGSPLSSPVFPR	21	sp|P17661|DESM_HUMAN
^a^Precursor proteins are named using UniProt entry names. AA: amino acid.			

### Functional verification of screened differentially expressed peptides

**Figure 5:**
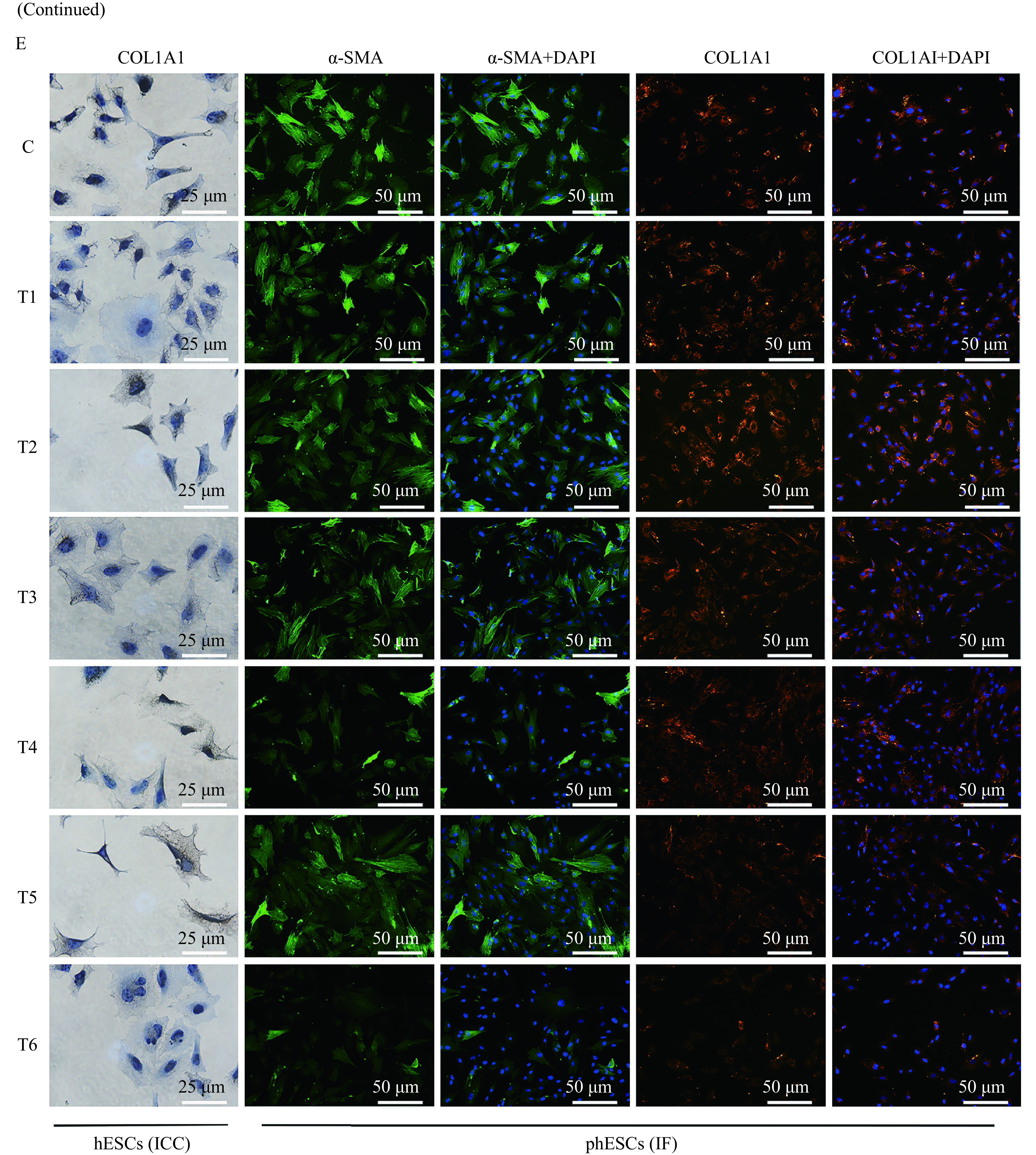
Gene and protein expression of fibrosis-related markers in hESCs or phESCs treated with different peptides.

The TGF-β1 (5 ng/mL) treated hESCs or phESCs were cocultured with T1, T2, T3, T4, T5, and T6 peptides at the concentration of 50 µmol/L, and the control group was cocultured with the same volume of peptide solvent sterilized water. After incubation for 48 hours, the total RNA of each group was extracted for RT-qPCR analysis. After incubation for 72 hours, the total protein of each group was extracted for Western blotting and the cells were selected for immunocytological experiment. The results showed that in the T6-treated hESCs group, the relative levels of mRNA expression of fibrosis-related genes such as *ACTA2* (α-SMA), *COL1A1*, *VIM*, *CDH2* (N-cadherin), and *FN1* (fibronectin) were significantly reduced compared with the control group. The relative expression of*CTGF* was decreased, but was not statistically significant (*P*>0.05) (***Fig. 5A***). In the T6-treated phESCs group, the mRNA expression of *ACTA2*, *VIM*, *CDH2*, and *CTGF* were significantly reduced compared with the control group, while the expression of *COL1A1* and *FN1* were also reduced but with no statistical difference (*P*>0.05) (***Fig. 5B***). T1 to T5 peptides did not decrease the expression of above fibrosis related genes (***Fig. 5A*** and ***B***). Among the six screened peptides, T6 had a significant and stable effect on reducing the protein expression of α-SMA (***Fig. 5C***) and COL1A1 (***Fig. 5C*** and ***E***) in hESCs, as well as in phESCs (***Fig. 5D*** and ***E***). T5 also reduced the protein expression of α-SMA and COL1A1, but with no statistical difference (*P*>0.05). No significant differences were also observed in T1, T2, T3, and T4 peptides treated group (***Fig. 5C***–***E***).

**Figure 6:**
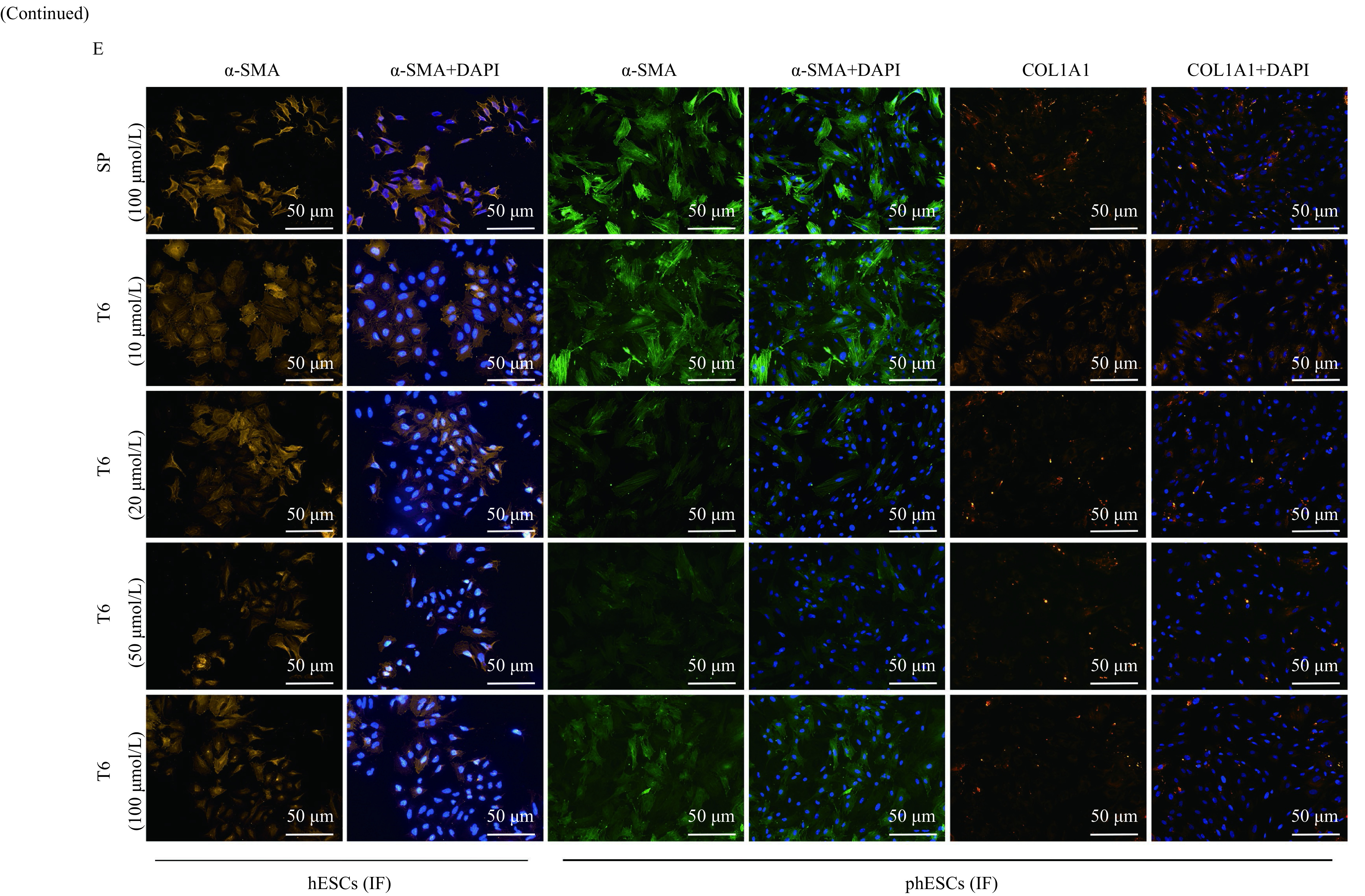
Effects of T6 on the gene and protein expression of fibrosis-related markers in hESCs or phESCs.

The TGF-β1 (5 ng/mL) treated hESCs or phESCs were cocultured with T6 (10 µmol/L, 20 µmol/L, 50 µmol/L, 100 µmol/L) and scrambled peptide (SP) (100 µmol/L). Compared with the SP treated group (peptide sequence: PGSGRFPGLSTLPFVAGPSPF), the relative mRNA expression levels of*ACTA2*, *COL1A1*, *CDH2*, *FN1* and *CTGF* were significantly downregulated both in hESCs and phESCs, and the relative mRNA expression level of *VIM* was also significantly downregulated in hESCs, but with not statistical difference in phESCs, when the T6 concentration was 50 μmol/L. Significant relative mRNA expression differences in individual indicators were observed when the T6 peptide concentration was 20 μmol/L or 100 μmol/L (***Fig. 6A*** and ***B***).

Compared with the SP treated group, the relative protein expression of α-SMA significantly decreased in the T6-treated hESCs at the concentration of 10 μmol/L, 50 μmol/L (*P*<0.05), and 100 μmol/L (*P*<0.01) (***Fig. 6C***), and in the T6-treated phESCs at the concentration of 20 μmol/L (*P*<0.05), 50 μmol/L (*P*<0.01), and 100 μmol/L (*P*<0.05) (***Fig. 6D***) by Western blotting. The relative protein expression of COL1A1 also significantly decreased in the T6-treated hESCs at the concentration of 50 μmol/L (*P*<0.05) (***Fig. 6C***), and in the T6-treated phESCs at the concentration of 20 μmol/L and 50 μmol/L (*P*<0.05) (***Fig. 6D***) by Western blotting. The protein expression of α-SMA and COL1A1 were decreased after treatment with T6 at the concentration of 10 μmol/L, 20 μmol/L, 50 μmol/L, and 100 μmol/L, while at the concentration of 50 μmol/L, the decrease was most obvious in both phESCs or hESCs (***Fig. 6E***). It was found that T6 could inhibit the expression of fibrosis related genes and proteins most significantly in hESCs and phESCs at a concentration of 50 μmol/L.

## Discussion

With the development of peptidome, endogenous peptides have become a new focus of pharmaceutical research. This study evaluated endogenous peptides with differential expression in IUA tissues and endometrial tissues during the normal proliferative stage and explored the formation and prevention of IUA from the standpoint of endogenous peptides in the hope of offering new perspectives and ideas for the diagnosis and treatment of IUA.

Most peptide functions are related to their precursor proteins. For example, T1 is derived from mimecan. It was reported that at the stage of fibrosis and calcification, mimecan was identified as having increased levels in the sample of stable atherosclerotic plaques^[25]^. Lumican, which is the precursor of T2, has been shown to be involved in collagen fibrillogenesis in extra-hepatic tissues^[26]^. Besides, desmin, which is the precursor of T6, was considered a fibrosis marker like α-SMA^[27]^. We also found that some of the precursor proteins of differentially expressed peptides were related to fibrosis^[28]^, and extracellular signal-regulated kinase (ERK) was related to the TGF-β1 signaling pathway; heat shock protein (HSP) was related to the vascular endothelial growth factor (VEGF) signaling pathway; collagen and thrombospondin 1 (THBS1) were related to ECM receptor interaction. These findings suggest that the differentially expressed peptides present in these fibrosis-related proteins may be involved in the formation of IUA. At the same time, 101 differentially expressed peptide sequences from 48 precursor proteins were located on the functional domain, indicating potential functions. A major concern, however, is that the vast majority of the identified 'peptides' are hemoglobin. We considered that it was related to the composition of the specimen. The adhesive tissue did not contain blood vessels, while the normal full-thickness endometrial tissue specimen contained blood vessels and blood components. So, there were significant differences in expression in the results. We believed that these proteins and peptides had no antifibrotic function.

Through bioinformatics analysis, the differentially endogenous peptides whose specific expression increased or decreased were further screened, and six endogenous peptides were selected. The peptides were cocultured with TGF-β1 and hESCs or phESCs. The effects of endogenous peptides on inhibiting fibrosis were evaluated at gene and protein levels. Among the six peptides screened, T6 had a significant and stable effect. It was found that the expression of T6 in IUA tissues was upregulated rather than downregulated during the early screening process. Because tissue repair had been completed and tissue fibrosis had been formed when the adhesive tissue was taken, it was considered that T6 is a compensatory upregulation of the response in the IUA tissues taken earlier. It was a consequence of IUA, but not the cause. Combined with the results of our experiment, it was further suggested that T6 could inhibit fibrosis rather than promote it.

During the occurrence and development of tissue fibrosis, the key effector cells of tissue fibrosis are fibroblasts and myofibroblasts, and these key effector cells can produce a large number of collagen components that constitute ECM, such as collagen Ⅰ and collagen Ⅲ^[29]^. Moreover, a variety of cytokines are involved in the pathological process of tissue fibrosis, among which TGF-β is the most important cytokine. As a multifunctional cell growth factor regulating cell proliferation and differentiation, TGF-β can directly promote the activation of *in situ* fibroblasts or stimulate the massive proliferation of myofibroblasts and excessive synthesis and deposition of ECM through endothelial-mesenchymal transformation and epithelial mesenchymal transformation. When TGF-β is continuously activated by injury, signaling pathways such as Smad signaling pathway, mitogen-activated protein kinase (MAPK) signaling pathway, and Wnt/β-catenin signaling pathway are activated, leading to the development and progression of fibrosis^[30]^. Therefore, TGF-β is an important target for the treatment of IUA.

In this study, hESCs were induced by TGF-β1, and a fibrotic cell model was constructed consistent with the literature reports, as evidence has shown that TGF-β1 induced the fibrosis of hESCs in a concentration and time dependent manner^[9]^. Therefore, in this experiment, a concentration of 5 ng/mL was selected for TGF-β1, and time points of 48 hours or 72 hours were selected to evaluate the effect of peptides on the inhibition of TGF-β1-induced hESCs and phESCs fibrosis. At the same time, this study also found that the effect of T6 on the inhibition of hESCs and phESCs fibrosis is related to the concentration of peptides, but not in direct proportion. It was suggested that T6 at a concentration of 50 µmol/L could achieve the best effect on inhibiting hESCs and phESCs fibrosis, although the relative mRNA expression of VIM and FN were puzzling in hESCs treated by T6 at the concentration of 20 µmol/L. T6 can dose-dependently inhibit TGF-β1-induced hESCs fibrosis, and it is speculated that T6 may be involved in the fibrosis process through TGF-β1 classical or nonclassical signaling pathways or other fibrosis-related pathways. However, the effect of inhibiting fibrosis in tissues still needs to be further verified by subsequent studies.

There are also some limitations in this study. The stability of the peptide was not studied. Although peptide drugs have high biological activity, strong specificity, good affinity with receptors, low toxicity, and little damage to human body, their disadvantages are poor stability and easy degradation *in vivo*^[17]^. Although we have chemically modified the peptides and verified their effectiveness through cell experiments, their stability still needs to be discussed. The specific mechanism of fibrosis of hESCs caused by T6 has not been clarified. In this study, only *in vitro* experiments were carried out, and their effectiveness needs to be further verified in animal experiments.

In conclusion, this study explored the peptide profiling of differentially expressed endogenous peptides in IUA samples from a new perspective. In this study, through bioinformatics analysis, the endogenous peptides differentially expressed in IUA and normal intimal tissues were further screened and six endogenous peptides were selected. Synthesized peptides were cocultured with TGF-β1 and hESCs or phESCs. It was verified that T6 could inhibit TGF-β1-induced hESCs and phESCs fibrosis *in* *vitro*. So far, no effective treatment method is available for IUA. The prevention and treatment of IUA remains a great challenge. This study may provide a new target for further clarifying the formation and effective treatment of IUA.
